# Effects of Gratitude Journaling on Patients with Breast Cancer: A Randomized Controlled Trial

**DOI:** 10.3390/curroncol32070400

**Published:** 2025-07-12

**Authors:** Minjeong You, Eunjung Kim

**Affiliations:** 1 Hwasun Chonnam National University Hospital, Hwasun, Jeollanam-do 58128, Republic of Korea; dn02861@cnuh.com; 2 Department of Nursing, College of Health Sciences, Honam University, Gwangju 62399, Republic of Korea

**Keywords:** breast cancer, nursing intervention, gratitude, journaling

## Abstract

Gratitude journaling is a simple and effective way to improve emotional well-being. However, its impact on people with breast cancer in South Korea has not been clearly understood. This study explored how writing a gratitude journal can help patients with breast cancer feel more grateful, resilient, and satisfied with life. Sixty patients from a university hospital in Jeollanam-do were randomly assigned to either a gratitude journaling group or a control group. The journaling group received guidance and wrote at least ten journal entries over three weeks, with weekly phone check-ins. The control group received no intervention. Before and after the program, the participants completed surveys. The results showed that those who kept gratitude journals had higher levels of gratitude, resilience, and quality of life than those who did not. These findings suggest that gratitude journaling can be a valuable and easy-to-use nursing strategy to support the emotional health of breast cancer patients.

## Simple Summary

Gratitude journaling means regularly writing down things you are thankful for. It is known to improve mood and emotional health. This study looked at whether keeping a gratitude journal could help breast cancer patients in South Korea feel more positive and better cope with their illness. In a randomized controlled trial, sixty patients at a university hospital were divided into two groups. One group wrote in a gratitude journal for three weeks, while the other group did not. The group who wrote journals felt more thankful, showed stronger resilience, and had a better quality of life than those who did not. This means that writing about gratitude may be a simple and helpful way to support the emotional well-being of people facing cancer. It could be used as an easy, low-cost tool in hospitals to help patients feel stronger and more hopeful.

## 1. Introduction

Breast cancer—the most commonly diagnosed cancer worldwide—accounts for approximately 24.2% of all cancers in women, making it the most prevalent malignancy in this group. In South Korea, breast cancer is the most frequently diagnosed cancer among women, with 29,528 new cases reported in 2022, representing 10.5% of all cancer cases [1]. Notably, 12.8% of patients with breast cancer in Korea are under the age of 40 years, a rate more than twice as high as that observed in Western countries [1].

In South Korea, advancements in early detection and treatment have led to a five-year survival rate of 93.8% among patients with breast cancer [2]. Given the high survival rates and the rising proportion of younger breast cancer patients, improving the quality of life (QoL) among survivors has emerged as a critical aspect of cancer care [3].

Breast cancer is no longer perceived as a terminal illness but rather as a chronic condition requiring continuous management and care [4]. Therefore, improving the breast cancer survivors’ QoL and alleviating their psychological distress is essential [3].

Women diagnosed with breast cancer often experience significant psychological distress throughout the treatment process, thereby increasing their risk of anxiety, depression, suicidal ideation, and neurocognitive impairments [5]. Such psychological distress can adversely impact not only survival rates but also overall health outcomes [6]. Thus, early identification and effective management of mental health issues among breast cancer survivors are crucial [7]. Furthermore, mental health is influenced more by an individual’s interpretation and perception of events than by the events themselves, making it a key determinant of personal happiness and overall QoL [5]. Consequently, various psychological interventions are required to enhance the mental well-being of patients with breast cancer. Among these, gratitude has acquired significant attention as an effective strategy to promote positive emotions [8].

Gratitude is defined as the psychological state of recognizing and appreciating the benefits received from others or the environment, leading to feelings of gratefulness and happiness [9]. It has been shown to help individuals reinterpret negative events in a positive manner and mitigate negative emotions [10]. Gratitude can also manifest as a dispositional trait, referred to as gratitude disposition, wherein individuals consistently experience and express appreciation for the kindness or support they receive from others [11]. Individuals with higher levels of gratitude disposition tend to engage in self-reflection during the process of recognizing gratitude, leading to lower levels of depression and stress and higher levels of happiness [9]. Additionally, gratitude tendencies have a positive impact on quality of life [12]. Gratitude tendencies can help individuals recognize and acknowledge positive life experiences even in uncertain situations related to health, potentially improving patients’ quality of life [13].

Gratitude journaling is an effective method to enhance emotional well-being by encouraging individuals to recognize reasons for gratitude in their daily lives, thereby increasing happiness and fostering a grateful mindset [14]. It is a writing-based intervention that systematically cultivates gratitude through intentional reflection on positive experiences, thus making it a highly effective and self-directed practice [15]. Grateful patients have better resilience, and cultivating positive emotions related to gratitude also enhances resilience [16].

Resilience refers to the psychological and social capacities of individuals to overcome adversity and grow from challenging experiences. Resilience is particularly crucial for patients with cancer, as it serves as an essential internal factor in maintaining QoL throughout treatment and survivorship [17]. Highly resilient individuals demonstrate a proactive and adaptive approach to stress, enabling them to recover from difficulties and exhibit enhanced coping abilities post-adversity [18]. Therefore, interventions aimed at fostering resilience among patients with cancer are essential. Cancer patients with high resilience exhibit fewer negative symptoms such as anxiety and depression and improve their quality of life by actively coping with the disease [19,20].

QoL includes subjective well-being across physical, psychological, social, economic, and spiritual domains and is a critical predictor of survival of patients with cancer [21]. The QoL of patients with breast cancer is significantly influenced not only by treatment side effects such as pain, nausea, and fatigue but also by a declines in physical and emotional functioning [22]. Therefore, identifying the factors that influence the QoL of patients with breast cancer in Korea and developing effective interventions to enhance their well-being are of paramount importance.

This study aimed to implement gratitude journaling as an accessible and effective intervention for patients with breast cancer, enabling them to engage in the practice regardless of time or location. By examining the effects of gratitude journaling on gratitude disposition, resilience, and QoL among breast cancer patients, this study sought to provide evidence for the use of gratitude journaling as an effective nursing intervention to improve the overall health and well-being of breast cancer survivors.

## 2. Methods

### 2.1. Study Design

This study used a randomized controlled trial (RCT) design with time-lagged groups and was conducted at the Chonnam National University Hwasun Hospital in South Korea. It compared gratitude disposition, resilience, and QoL between the experimental group, which practiced gratitude journaling, and the control group, which did not receive any specific intervention. To prevent intervention contamination, the control group was assessed first, followed by a gratitude journaling intervention for the experimental group (Table 1).

### 2.2. Participants

A convenience sample of patients who underwent primary treatment for breast cancer, including surgery or chemotherapy, and had not exceeded five years of adjuvant therapy was selected. Patients who understood the purpose of the study and voluntarily agreed to participate after providing a written informed consent were included in the study.

The sample size was calculated using G*Power 3.1.9.7 software for independent t-tests. Based on a significance level of 0.05, a large effect size of 0.80, and a power of 0.80, the minimum required sample size for a two-tailed independent *t*-test was 26 participants per group, totaling 52 participants. Considering a dropout rate of approximately 15%, a total of 60 participants were recruited, with 30 each assigned to the experimental and control groups.

The inclusion criteria were as follows: (1) women aged 18 years or above who were diagnosed with breast cancer, (2) the completion of primary treatment (surgery or chemotherapy) for the primary tumor within the past five years, and (3) understanding the purpose of the study and voluntarily agreeing to participate. The exclusion criteria were as follows: (1) patients with recurrent or metastatic cancer or other primary cancers, (2) individuals unwilling to participate fully in the study program, (3) patients with previous participation in similar research, (4) the inability to read Korean or communicate effectively, (5) the diagnosis of a mental illness, and (6) patients with other acute or chronic conditions, including cardiovascular, respiratory, or neurological diseases, uncontrolled hypertension or diabetes, or chronic renal failure.

### 2.3. Ethical Issue

This quasi-experimental study was conducted at Chonnam National University Hwasun Hospital in Korea. It compared gratitude disposition, resilience, and quality of life (QoL) between an experimental group that practiced gratitude journaling for three weeks and a control group that received no specific intervention. To prevent contamination of the intervention, the control group was assessed first, followed by implementation of the gratitude journaling intervention for the experimental group (Table 1). Data collection was conducted by a single trained nurse. A total of 60 patients who met the eligibility criteria participated in the study. The study was reported in accordance with the Consolidated Standards of Reporting Trials (CONSORT 2017) guidelines (see Supplementary Materials) and was approved by the Institutional Review Board of Chonnam National University Hwasun Hospital (approval number: CNUHH-2024-166). It was registered with the Korea Clinical Research Information Service (KCT0010522).

The authors confirm that all ongoing and related trials for this intervention have been registered. Notably, due to the nature of this intervention, ethical approval was obtained at the outset, but we recognized the importance of trial registration only after submitting the manuscript to *Current Oncology* and reviewing the World Health Organization (WHO) guidelines more carefully. Accordingly, the trial was successfully registered retrospectively with the appropriate permissions.

### 2.4. Randomization

The participants were randomly assigned numbers using a random function in Microsoft Office Excel. Subsequently, a random allocation software (www.randomizer.org) was used to allocate the participants into two groups based on their assigned random numbers. This allocation was conducted prior to any intervention, and the measurements collected immediately after group assignment served as the baseline assessment.

### 2.5. Research Procedure

#### 2.5.1. Pre-Test

Prior to the intervention, a pre-test was conducted using a self-reported questionnaire to assess the participants’ general characteristics, gratitude disposition, resilience, and QoL. The survey was conducted in a consultation room in the endocrine surgery ward of the hospital to protect the privacy of the subjects and create an environment where they could focus on completing the questionnaire. During the survey process, the researcher distributed the questionnaire, answered questions related to the questions, and provided any necessary assistance. In principle, the subjects were encouraged to fill out the questionnaire themselves, but if they had difficulty, the researcher read the questions and helped them respond. The questionnaire took about 10–15 min to complete, and the completed questionnaires were checked for missing responses, sealed in envelopes, and collected immediately.

#### 2.5.2. Intervention

##### Experimental Group

The intervention was conducted over a period of three weeks, during which the experimental group was instructed to write gratitude journals at least 10 times to help them form a habit. Based on the gratitude journal guidance method proposed by Jung [23], specific themes such as “Gratitude for Myself”, “Gratitude for My Family”, and “Gratitude for Nature and Objects” were assigned by day of the week. For the “Gratitude for Oneself” theme, the participants were asked to choose from the following prompts: (1) reflect on what life would be like without a specific body part and express gratitude for one’s physical body; (2) identify and appreciate one’s personal strengths; or (3) acknowledge one’s current circumstances (e.g., location, season, or era) and express gratitude for them.

For the “Gratitude for Family” theme, the participants selected a family member (e.g., father, mother, sibling, grandfather, aunt, or uncle) and wrote about why they were thankful for that person.

In the “Gratitude for Nature and Objects” theme, the participants were encouraged to imagine the absence of everyday natural elements (e.g., the sun, moon, stars, air, or salt) or common objects (e.g., chairs, desks, shoes, umbrellas, or toilets) and write about their appreciation for them.

Lastly, under the theme “Gratitude for People Around You,” the participants were asked to write about things they were thankful for in relation to people they encounter in daily life, such as friends, teachers, or supermarket employees.

The participants received training to ensure that they could write about each theme at least three times for a three-week period. They were asked to write about things they were grateful for at a convenient time during the day for approximately 15 min. In addition to identifying the subject of their gratitude, the participants were encouraged to briefly describe the reasons for their gratitude. Furthermore, they were provided with a book containing instructions on gratitude journaling, a dedicated notebook, and a pen.

**Figure 1:**
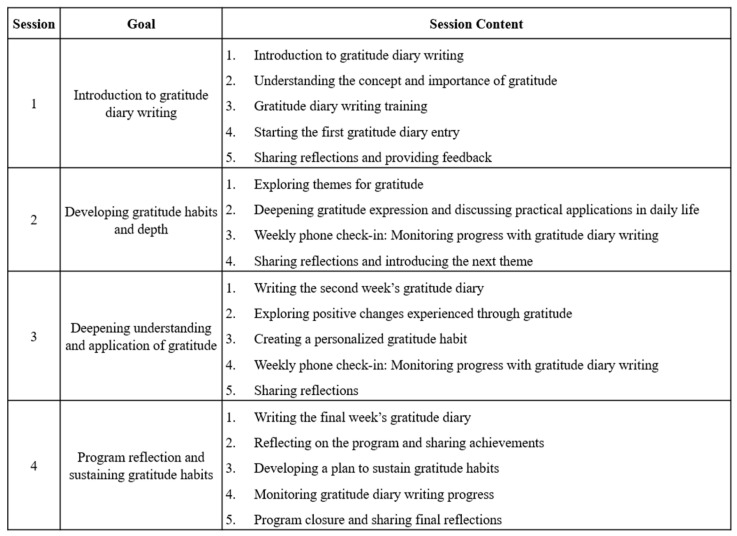
Structure and contents of gratitude journaling.

After the first journal entry, a check was conducted to ensure that the writing aligned with its intended purpose. Subsequently, the participants were monitored through weekly phone calls for three weeks to confirm that they had completed at least 10 journal entries (Figure 1).

##### Control Group

The control group did not receive any specific intervention during the three-week period. After three weeks, they completed the post-test and were provided with the same gratitude diary book, notebook, and pen as the experimental group. Additionally, after the experiment, participants in the control group were given the option to receive writing education on gratitude journaling, if they wished.

#### 2.5.3. Post-Test

After the intervention, a post-test was conducted using a self-report questionnaire to assess the participants’ gratitude disposition, resilience, and QoL.

### 2.6. Data Collection

Baseline data (pre-test) were collected in September 2024. Interventions were conducted between September and October. Post-test data were collected three weeks after the collection of the baseline data.

### 2.7. Measurement and Instruments

#### 2.7.1. Gratitude Disposition

Gratitude disposition was measured using the Korean version of the Gratitude Questionnaire-6 (K-GQ-6), originally developed by Mccullogh et al. [24] and translated and validated by Kwon, Kim, and Lee [25]. Approval for use of the scale was obtained from the original developer and the translator. The scale consists of six items rated on a 7-point Likert scale, with higher scores indicating a higher level of gratitude. Cronbach’s α was 0.85 in Kwon’s study [25] and 0.91 in this study.

#### 2.7.2. Resilience

Resilience was assessed using the Korean version of the Connor–Davidson Resilience Scale (K-CD-RISC), which was originally developed by Connor and Davidson [26] and translated and validated by Jeon and Lee [27]. Approval for use of the scale was obtained from both the original developers and the translator. This scale consists of 25 items categorized into five subscales: hardiness, persistence/endurance, optimism, support, and spirituality. Each item is rated on a 5-point Likert scale, with higher scores indicating greater resilience. Cronbach’s α was 0.89 at the time of development [26] and 0.96 in this study.

#### 2.7.3. QoL

QoL in patients with breast cancer was evaluated using the Functional Assessment of Cancer Therapy-Breast (FACT-B) scale, developed by the Functional Assessment of Chronic Illness Therapy (FACIT) group. The Korean version of the FACT-B was translated and validated by Yoo et al. [28], and approval for its use was obtained from the FACIT group. The scale comprises 37 items across five domains: physical well-being, social/family well-being, emotional well-being, functional well-being, and additional breast cancer-specific concerns. Each item is rated on a 5-point Likert scale, with higher scores indicating a better QoL. Cronbach’s α was 0.87 in Yoo et al.’s study [28] and 0.95 in this study.

### 2.8. Data Analysis

Data were analyzed using IBM SPSS Statistics for Windows version 28. Descriptive statistics, including means, standard deviations, and percentages, were calculated to summarize the characteristics of the study variables.

An independent *t*-test was conducted to assess the preliminary homogeneity of the study variables between the experimental and control groups. To evaluate the within-group changes before and after the intervention in the experimental group, a paired *t*-test was performed for gratitude disposition, resilience, and QoL. An independent *t*-test was conducted to examine differences between the experimental and control groups to assess the effects of the intervention on these variables.

A two-tailed significance level of *p* ≤ 0.05 was applied for all statistical analyses. The reliability of the measurement tools was assessed using Cronbach’s α. These statistical methods provided insights into the effectiveness of the intervention on the outcomes measured in this study.

## 3. Results

### 3.1. Demographic Characteristics of Patients

**Figure 2:**
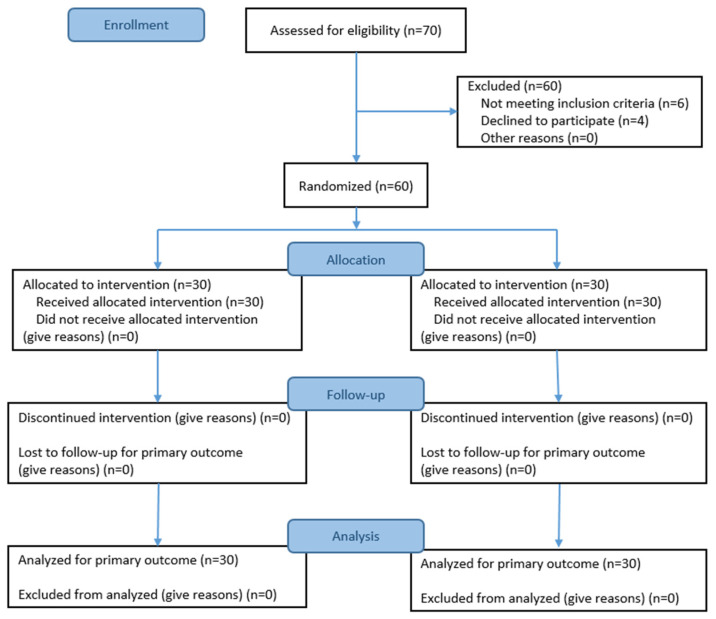
CONSORT2017 participant flow diagram with response rates.

The final number of participants in the study was 60, with 30 each in the experimental and control groups (Figure 2). Table 1 presents the general characteristics of the two groups before the intervention. No significant differences were observed between the groups in terms of age, marital status, education, economic satisfaction, religion, primary caregiver, cancer stage, or hormone therapy. Additionally, no significant differences were observed in terms of academic self-efficacy, learning engagement, or learning interest, confirming the homogeneity of the experimental and control groups before the intervention (Table 2).

### 3.2. Comparison of Gratitude Disposition, Resilience, and QoL

Table 3 presents the results of gratitude journaling for both the experimental and control groups. There were significant differences between the groups in terms of gratitude disposition, resilience, or QoL, either before or after the intervention.

In the experimental group, gratitude disposition significantly increased from 5.38 before the intervention to 6.22 after the intervention (*p* < 0.001). By contrast, the control group showed a significant decrease in gratitude disposition, from 5.07 before the intervention to 4.66 after three weeks (*p* = 0.007). The difference in gratitude disposition score between the two groups was statistically significant (*t* = 6.73, *p* < 0.001).

In the experimental group, resilience increased significantly from 2.57 before the intervention to 2.80 after the intervention (*p* = 0.004). However, no significant changes were observed in the control group pre- and post-intervention (*p* = 0.097). The difference in resilience score changes between the two groups was statistically significant (*t* = 3.20, *p* = 0.002).

In terms of QoL, the experimental group showed a significant improvement, with scores increasing from 2.41 before the intervention to 2.80 after the intervention (*p* < 0.001). By contrast, no significant changes were observed in the control group pre- and post-intervention (*p* = 0.898). The difference in QoL score between the two groups was statistically significant (*t* = 3.66, *p* = 0.001).

## 4. Discussion

This study conducted a gratitude journaling intervention for patients with breast cancer in Korea and examined its effects on gratitude disposition, resilience, and QoL. The findings indicate a positive effect of gratitude journaling on all three variables. Internet usage is increasing worldwide, and the number of cancer patients obtaining medical information through the Internet is also increasing [29]. In addition, online communities among social networking services (SNSs) enable people with common interests to connect with each other, and approximately 20% of cancer patients participate in online communities [30]. Patients with the same condition exchange disease-specific information, share experiences, and receive emotional support through online communities. In this study, a time-lagged randomized controlled design was used to minimize the spread of the experiment.

Previous studies have commonly utilized the gratitude journaling format developed by McCullough and Emmons [24]. In this method, participants are asked to spend 10–15 min writing about up to six things they are grateful for on a given day in a free-form style. However, in the Korean educational context, which emphasizes self-reflection and self-discipline, the unstructured format of McCullough and Emmons’ gratitude journaling may present difficulties, especially for individuals unfamiliar with expressive writing.

To address this, the present study adopted the structured gratitude journaling method proposed by Jung [23], which is considered more suitable for individuals who benefit from guided reflection. Rather than simply listing grateful experiences, Jung’s method encourages participants to write about gratitude-related topics by day and reflect on the personal meaning of each experience. This structured and introspective format is particularly appropriate for populations requiring emotional support, such as older adults, patients, and students.

Gratitude disposition significantly increased in the experimental group and significantly decreased in the control group. This indicates that participants who engaged in gratitude journaling experienced an enhancement in their gratitude disposition, while those in the control group, who did not receive any specific intervention, showed a decline after three weeks. This decrease may be attributed to the pain and side effects commonly experienced following chemotherapy or surgery. This aligns with previous research indicating that patients with cancer undergoing chemotherapy experience depression levels five-times higher than those of the general population [31]. Furthermore, these findings are consistent with those of previous studies, including one conducted in Poland, where breast cancer patients who wrote gratitude journals for two weeks—listing reasons for gratitude—showed improvements in everyday psychological functioning and coping abilities [21]. Similarly, a study involving African-American breast cancer patients in the United States reported increased levels of gratitude after writing gratitude journals at least twice per week over an eight-week period [32]. These results suggest that gratitude journaling may serve as an effective psychological intervention for breast cancer patients experiencing high levels of stress, regardless of race, age, or cultural background.

Although gratitude is generally considered a stable trait rather than one prone to sudden change, breast cancer patients often face persistent physical pain, psychological stress, and emotional fatigue that can lower their baseline gratitude levels compared to the general population [33]. These individuals may struggle to recall or recognize positive experiences due to the overwhelming nature of their illness and treatment. Despite the short intervention period of only three weeks, the active process of reflecting and recording gratitude appeared to amplify positive emotions, enhance daily functioning, and contribute to an increase in gratitude levels. Given the chronic nature of breast cancer and the potential for extended treatments such as hormone therapy, future longitudinal studies should examine the long-term relationship between gratitude journaling and sustained gratitude levels over periods exceeding one year.

Resilience was also higher in the experimental group than in the control group after the gratitude journaling intervention. Although there is a lack of direct comparative studies, this finding is consistent with that of studies showing increased resilience in upper elementary school students after gratitude journaling [34]. Patients with cancer often experience diminished resilience because of stressors such as cancer diagnosis, risk of recurrence, and treatment-related side effects from surgery, chemotherapy, and radiation therapy [35]. Resilience is a psychological mechanism that helps individuals adapt flexibly to adversity. In patients with cancer, resilience plays a crucial role in alleviating psychological distress and improving QoL during disease progression [36]. Therefore, it is essential to foster resilience based on the patient’s psychological state and environmental factors.

Finally, QoL significantly improved in the experimental group. This finding is consistent with that of previous studies showing that self-compassion-related writing interventions improved QoL in patients with breast cancer [33]. By contrast, the control group, which did not receive any intervention, experienced a slight decline in QoL after three weeks. This result aligns with that of previous studies reporting that 82.3% of patients with cancer had low QoL scores and that 54.4% experienced severe depression, which adversely impacted their psychological well-being [37]. As the incidence of breast cancer in younger populations increases and survival rates improve, various physical, psychological, and social challenges faced by breast cancer survivors may contribute to a decline in their QoL. The results of this study suggest that gratitude journaling is an effective intervention to enhance the QoL of patients with breast cancer. These findings highlight the need for proactive interventions to address the challenges faced by patients with breast cancer and improve their overall well-being.

### Limitations and Strengths

This study has a few limitations. First, it was conducted at a single university hospital in a specific region of South Korea and included only patients with breast cancer. Therefore, generalizing the findings to a broader population of patients with cancer may be challenging. Future studies should recruit participants with various types of cancer from multiple hospitals to improve the external validity and generalizability of the findings.

Second, although a power analysis was conducted to determine the sample size, further research with a larger and more diverse sample is needed to better reflect the variation of experiences among breast cancer patients. Moreover, although the gratitude journaling intervention in this study was implemented for at least 10 sessions over three weeks, future studies should explore different frequencies and durations of the intervention, as well as conduct long-term follow-up evaluations to assess the sustainability of its effects.

Additionally, a future study design should consider including an active control group that engages in non-directive reflection exercises. This would help clarify whether the observed outcomes are attributable to the specific focus on gratitude or to differences in attention and support.

Third, a wider range of methodological approaches is needed. Future studies should incorporate objective biological or behavioral indicators in addition to self-reported psychological measures to provide a more comprehensive evaluation of intervention effects. Furthermore, the quantitative nature of this study limits its ability to capture the diversity of patient experiences. Human-centered qualitative or mixed-methods research is recommended for future studies to better understand individual perspectives and contextual factors.

Fourth, in addition to the psychological indicators used in this study, future research should include physiological or clinical outcomes, which would allow for a more holistic assessment of the intervention’s benefits.

Conversely, a key strength of this study is that it is the first to apply gratitude journaling among breast cancer patients in various settings within the Korean context. This simple, non-invasive, and cost-effective intervention yielded positive outcomes by enhancing gratitude disposition, resilience, and quality of life. Furthermore, gratitude journaling holds promise as a supportive strategy not only for breast cancer patients but also for individuals with other types of cancer and chronic illnesses, contributing to emotional well-being and psychological stability.

## 5. Conclusions

Gratitude journaling among patients with breast cancer increased their gratitude disposition and resilience, ultimately improving their QoL. Consequently, this study provides foundational data supporting the positive effects of gratitude journaling on patients with breast cancer and its effectiveness as a nursing intervention for cancer patients. Implementing a gratitude journaling intervention after simple education can significantly enhance the psychological recovery of patients with cancer.

## Figures and Tables

**Table 1:** Research design.

Group	Pre-Test	Intervention	Post-Test	Pre-Test	Intervention	Post-Test
Control group	C1	-	C2			
Experimental group				E1	X1	E2

**Table 2:** Comparison of demographic characteristics of patients between groups.

	Categories	Exp. (n = 30)	Cont. (n = 30)	*χ*^2^ or *t*	*p*
		n (%)	n (%)		
Age	≤39 years	2 (6.7%)	3 (10.0%)	5.12	0.163
	40–49 years	8 (26.7%)	15 (50.0%)		
	50–59 years	10 (33.3%)	8 (26.7%)		
	≥60 years	10 (33.3%)	4 (13.3%)		
Marital status	Single	2 (6.7%)	2 (6.7%)	1.08	0.584
	Married	25 (83.3%)	27 (86.7%)		
	Divorced/separated	3 (10.0%)	1 (3.3%)		
Education level	High school	9 (30.0%)	10 (33.3%)	1.59	0.663
	University or higher	21 (70.0)	20 (66.7%)		
Economic satisfaction	Dissatisfied	1 (3.3%)	5 (16.7%)	3.34	0.188
	Neutral	24 (80.0%)	19 (63.3%)		
	Satisfied	5 (16.7%)	6 (20.0%)		
Religion	No	10 (33.3%)	15 (50.0%)	3.77	0.439
	Yes	20 (66.7%)	15 (50.0%)		
Primary caregiver	Spouse	12 (40.0%)	16 (53.3%)	3.04	0.551
	Child	6 (20.0%)	6 (20.0%)		
	Sibling or relative	7 (23.3%)	5 (16.7%)		
	Friends	5 (16.7%)	3 (10.0%)		
Cancer stage	Stage 0	1 (3.3%)	0 (0.0%)	3.94	0.268
	Stage 1	13 (43.3%)	9 (30.0%)		
	Stage 2	10 (33.3%)	17 (56.7%)		
	Stage 3	6 (20.0%)	4 (13.3%)		
Hormone therapy	No	17 (56.7%)	21 (70.0%)	1.15	0.284
	Yes	13 (43.3%)	9 (30.0%)		

**Table 3:** Comparison of gratitude disposition, resilience, and QoL of patients between the groups.

Variables	Groups	Pre-Test	Post-Test	*t*	*p*	Difference	*t*	*p*
		Mean ± SD	Mean ± SD			Mean ± SD		
Gratitude disposition	Exp. (*n* = 30)	5.38 ± 0.98	6.22 ± 0.77	−6.94	0.000	0.83 ± 0.66	6.73	0.000
	Cont. (*n* = 30)	5.07 ± 1.02	4.66 ± 1.09	2.92	0.007	−0.41 ± 0.77		
Resilience	Exp. (*n* = 30)	2.57 ± 0.48	2.80 ± 0.38	−3.15	0.004	0.23 ± 0.40	3.20	0.002
	Cont. (*n* = 30)	2.49 ± 0.73	2.31 ± 0.68	1.72	0.097	−0.18 ± 0.59		
QoL	Exp. (*n* = 30)	2.41 ± 0.52	2.80 ± 3.35	−5.26	0.000	0.39 ± 0.40	3.66	0.001
	Cont. (*n* = 30)	2.35 ± 0.72	2.34 ± 0.73	0.13	0.898	−0.01 ± 0.44		

## Data Availability

The datasets generated and analyzed during the current study are available from the corresponding author upon request.

## Supplementary Materials

The following supporting information can be downloaded at: https://www.mdpi.com/article/10.3390/curroncol32070400/s1, Table S1: CONSORT Statement for Randomized Trials of Nonpharmacologic Treatments: A 2017 Update and a CONSORT Extension for Nonpharmacologic Trial Abstracts.

## Abbreviation

The following abbreviations are used in this manuscript:

QoL	Quality of Life
